# Wastes as support materials for the development of biological barriers for landfill liners

**DOI:** 10.1007/s11356-026-37663-6

**Published:** 2026-03-25

**Authors:** Alice Kimie Martins Morita, Mercedes Regadío

**Affiliations:** 1 Department of Geology, Physics, and Inorganic Chemistry, Universidad Rey Juan Carlos, Móstoles, Spain; 2 Department of Geology and Geochemistry, Universidad Autónoma de Madrid, Madrid, Spain

**Keywords:** Construction and demolition waste, Tire waste, Biofilms, Leachate, Municipal landfill, Circular economy

## Abstract

Biological clogging permits reducing the permeability of waste materials, which can be used in the design of barriers for retaining contaminants, promoting environmental protection and circularity. The present study evaluated using construction and demolition waste (CDW) and tire waste (TW) as support materials for developing biobarriers within the landfill design. The experimental setup consisted of vertically positioned PVC columns, filled with CDW, its mixture with tire waste (CDW/TW) or with anaerobic biomass (in CDW). The columns were fed with raw leachate collected from an active municipal landfill in Madrid, Spain, (dissolved organic carbon (DOC) 278 ± 150 mg/L, pH 7.4 ± 0.4, electrical conductivity (EC) 14,013 ± 5218 µS/cm, oxidation–reduction potential (ORP) –126 ± 100 mV). Head loss and flow measurements enabled calculating the permeability of the materials over time (Darcy’s law). Inlet and outlet samples were collected daily (pH, EC, ORP, temperature) and biweekly (cations, anions, trace elements, DOC). The results showed a reduction in hydraulic conductivities of 2–3 orders of magnitude, achieving better results for the CDW/TW columns (10^–7^ m/s in 170 days), possibly due to the hydrophobicity of tire, enhancing biofilm adhesion. An exponential correlation showed that hydraulic conductivities of 10^–9^ m/s (the minimum legal limit for compacted clay liners under waste landfills) can be reached before 420 days of operation. Physicochemical analysis indicated sulfate reduction, possibly contributing to the precipitation of heavy metals into sulfide minerals within the biobarrier. The incorporation of waste materials in landfill liners can reduce contamination in the medium and long terms and contribute to the circular economy.

## Introduction

Solid waste final disposal can cause severe impacts on the environment and public health, including soil and water contamination by leachates (Huang et al. 2024). Even in modern sanitary landfills, the installed engineered barriers showed decreasing hydraulic performance after 8 years of landfill operation, which can enable leachate leakage and environmental contamination in the medium and long terms (Sun et al. 2019).

This scenario is worrisome, considering the forecast of waste generation increase—70% by 2050, according to Kaza et al. (2018)—and the number of old landfills that are abandoned and continue to contaminate the surroundings. In Europe, for example, there are 150,000 to 500,000 landfills, from which 90% are old unlined ones that will soon require remediation measures (Vaverková, 2019). Additionally, the projected landfills rely on containment barriers made of non-renewable sources (clays and geomembranes), which are still associated with a linear economy and can generate high economic and environmental costs when such resources are not locally available.

Therefore, it is necessary to develop barriers effective in the long term and made of renewable, recycled and/or locally available materials, which can be easily adapted to different socioeconomic contexts. Biological barriers (biobarriers) have been applied either to the remediation (Budania and Dangayach 2023; Upadhyay and Sinha 2018) or the containment of contaminated groundwater (James et al. 2000), but there are few studies evaluating their performance in landfill liners. In this regard, biological clogging in leachate collection systems has been approached as a problem and not as a possibility to be exploited (Touze-Foltz et al. 2021; Van Gulck and Rowe 2004).

Nevertheless, some studies showed that biological clogging could be advantageous when designing barriers. Safari and Valizadeh (2017) indicated that the design approach of liners should be changed to a performance-based approach, so that biological clogging causing permeability reduction should be incorporated, permitting a reduction in the clay liner width and thus the costs and use of resources. Zhang et al. (2021) observed that soils following bacterial treatment (inoculation with *Escherichia coli*) could reach values of hydraulic conductivities low enough for barrier applications. Tang et al. (2018) concluded that biological clogging could be used in landfill engineering to reduce the permeability of liner materials, increasing the breakthrough time and enhancing the barrier performance.

Thus, it is suggested that biological clogging can also be used to obstruct different kinds of materials, including waste, which could be used as support to grow biofilms in the design of biobarriers. On this matter, in the last years, different types of waste have been incorporated in the promotion or improvement of remediation and environmental processes, generating lower cost/higher benefit and contributing to the circular economy, by valorizing production waste that are commonly disposed of in landfills (Ghandehari et al. 2023; Sanchez-Ramos et al. 2023; Rubinos and Spagnoli 2018). Additionally, some waste materials can enhance biofilm adhesion (Miao et al. 2020; Germec et al. 2020; Pereira et al. 2021), which would be beneficial for the construction of biobarriers. Thus, the use of production waste in the development of barriers, especially through the incorporation of biologically regulated processes, could provide a solution to two environmental problems: (a) soil and groundwater contamination, and (b) the dominant linear economy, which still causes a large proportion of waste to end up in landfills. Additionally, it has been reported that biofilms can largely remain intact and continue to reduce hydraulic conductivities even in extreme conditions of pH (3 to 9) and salinity (5 to 20 g/L), which theoretically guarantee the performance of such barriers (Zhang et al., 2018).

The present research aimed to evaluate the use of waste generated in high quantities globally—construction and demolition waste and tire waste—as support materials for the development of biological barriers within the landfill design. Organic matter, nutrients, and bacteria naturally present in leachates could permit the biofilm maintenance over time, and the development of this biofilm would not impact the geotechnical or structural characteristics of the landfill (Gyr 1998). It is expected that such support materials could facilitate biofilm formation and the deposition of solids and salts, which would reduce the permeability of the materials and enable them to function as barriers in landfill design (Morita et al., 2022).

**Fig. 1:**
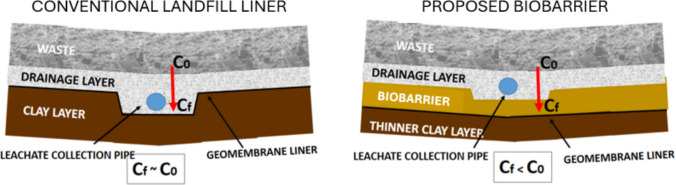
Conceptual scheme of the proposed biobarrier integrated into a landfill liner system, positioned above the compacted clay liner and geomembrane as part of a multi-barrier containment approach

The proposed biological barrier (biobarrier) is intended to be incorporated within the landfill liner system, as an intermediate layer above the compacted clay liner and the geomembrane, functioning as part of a multi-barrier containment strategy (Fig. 1). In this configuration, leachate percolating from the waste body first passes through the biobarrier, where biological clogging, physicochemical precipitation, and sorption processes contribute to a progressive reduction in hydraulic conductivity and contaminant attenuation. By decreasing the hydraulic and contaminant loads reaching the underlying engineered liners, the biobarrier is expected to enhance their long-term performance and durability, while not replacing but complementing conventional liner material.

## Materials and methods

The experimental set-up was composed of 26 columns made of PVC (approx. 50 mm-diameter × 600 mm-length), which were filled with waste and their mixtures: 8 columns were filled with construction and demolition waste, < 2 mm particle size (“CDW”); 8 columns with a mixture of CDW and tire waste (TW), 50% v/v (“CDW/TW”); and 8 columns with CDW inoculated with anaerobic sludge obtained from a digester treating domestic wastewater in Madrid, Spain (700 g TS/L, 12 g VS/L) (“IN CDW”). All the columns were fed with real leachate obtained from an active landfill in Madrid (Spain) whose physicochemical characteristics are shown in Table 1. Other 2 columns were filled with CDW and were fed with tap water, functioning as a flux control (“Blank”).

**Table 1:** Physicochemical parameters of the raw leachate used in the present study. World Health Organization drinking standards (WHO, 2022) are also presented

Parameter	Mean ± standard deviation			Standards WHO (2022)
	**Specified units**	**mg/L**	**µg/L**	
**pH**	7.5 ± 0.4			
**EC (µS/cm)**	14,013 ± 5218			
**ORP (mV)**	−126.1 ± 100.1			
**DO**		1.2 ± 0.9		
**Alkalinity (mgCaCO**_**3**_**/L)**		4923.4 ± 1939.0		
**DOC**		278.2 ± 150.5		
**Cl**^**−**^		1303.4 ± 383.6		
**Na**		1117.8 ± 260.9		50,000
**K**		574.1 ± 3.6		
**Mg**		232.5 ± 4.8		
**Ca**		196.6 ± 48.7		
**SO**_**4**_^**2−**^		78.9 ± 48.8		
**NO**_**3**_^**−**^		38.2 ± 38.1		50,000
**P**		0.8 ± 0.5		
**NH**_**4**_		0.05 ± 0.03		
**Fe**			605.3 ± 251.0	
**Mn**			340.7 ± 69.3	80
**Ba**			252.5 ± 82.2	1300
**As**			225.7 ± 2.8	10
**Ni**			75.9 ± 31.0	70
**Zn**			56.2 ± 79.5	
**Cr**			32.9 ± 9.8	50
**Co**			16.6 ± 9.1	
**Pb**			0.4 ± 0.5	10

The 50% v/v mixing ratio was selected as an exploratory proportion commonly adopted in preliminary composite material assessments, aiming to maximize surface heterogeneity while maintaining sufficient mechanical stability and compaction. This ratio was not previously optimized, and a systematic evaluation of different mixing proportions is suggested as the focus of future studies.

The experimental design included multiple columns per treatment not only to ensure replicability but also to allow operational flexibility during long-term testing. Columns were operated independently and could be stopped at different times for inspection or verification purposes while maintaining a sufficient number of active replicates throughout the experiment. Blank columns were intended as qualitative controls to verify system stability and potential material leaching rather than for statistical comparison and therefore were operated with a reduced number of replicates.

Each column had 3 piezometer ports (in the beginning, at 300 and 600 mm) to measure the hydraulic heads. Head loss was measured between pressure ports located immediately at the entrance and exit of the porous medium, downstream of the inlet and upstream of the outlet fittings; therefore, the calculated hydraulic gradient reflected only the resistance of the packed material. Hydraulic conductivity calculations over time were based on Darcy’s law, which depends on the measured flow rate, hydraulic gradient, and cross-sectional area. Reynolds numbers calculated based on pore velocity and effective particle size remained below unity, confirming laminar flow conditions and the applicability of Darcy’s law throughout the experiment.

The CDW and TW used in this study comply with most limits established in the European Decision 2003/33/CE for being considered inert materials, but attention should be given to the leaching of cadmium (Cd) and antimony (Sb) from CDW, and the particulate organic matter content from TW (Regadío et al. 2023). However, the leaching of these elements would be highly restricted, as the intended application—primarily a bottom barrier—maintains reducing conditions that render these elements essentially immobile.

Regarding particle size distribution, Regadío et al. (2023) obtained the following results for the same CDW used in the present study: D50 = 0.60 mm, D30 = 0.33 mm, D10 = 0.10 mm, external specific surface 4.29 ± 0.25 m^2^/g. Because of its texture and textile nature, such analysis could not be conducted for TW.

The materials and mixtures were compacted inside the columns considering the optimal conditions obtained in Proctor tests (British Standards Institution, 1990), adapted to the dimensions of the PVC columns. In this regard, 10% moisture content was adopted for all barrier configurations, and porosities of 7% were obtained for CDW and IN CDW columns and 12% for CDW/TW columns. Such porosity values were determined gravimetrically by measuring the volume of water required to fully saturate the compacted material at the beginning of the experiment. This method yields the total connected porosity of the compacted medium, which can be relatively low for fine-grained, highly compacted materials used in liner applications. Although the porosity is low, continuous flow was observed throughout the experiment, indicating that interconnected pore spaces remained available for leachate percolation.

**Fig. 2:**
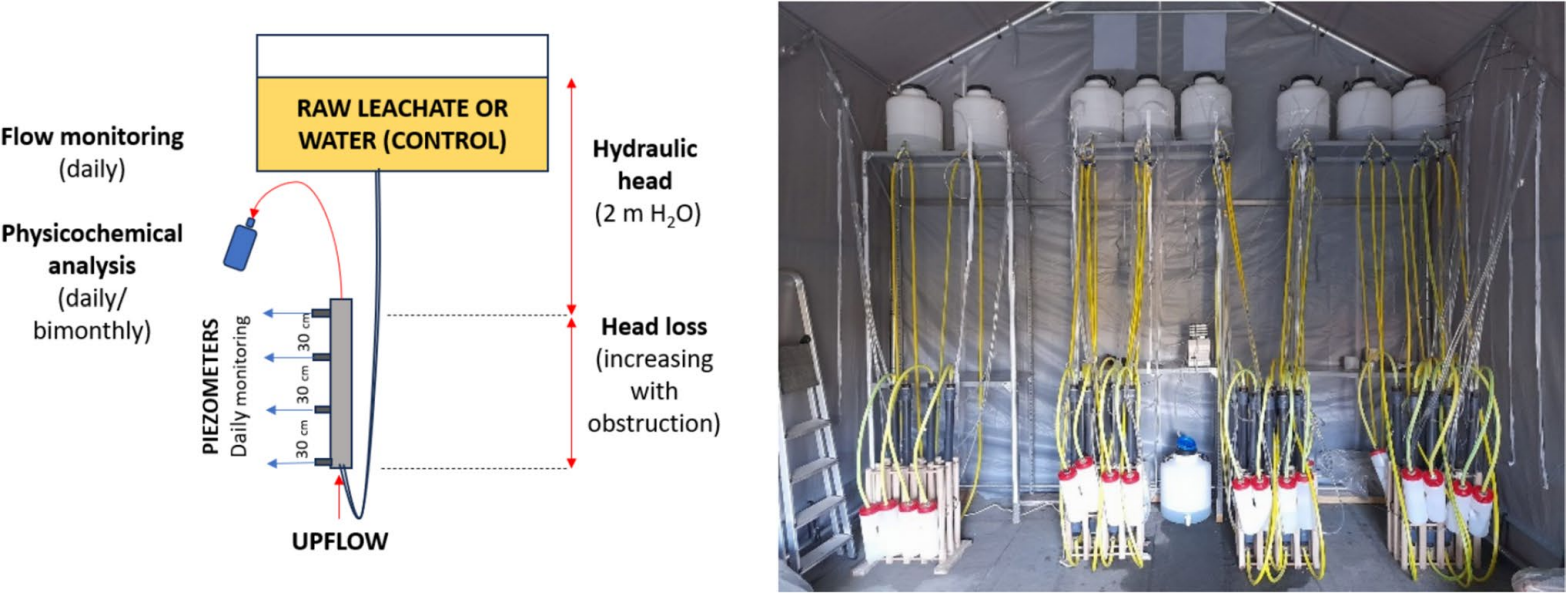
Scheme of permeability test performed with the column barriers (left) and experimental setup (right)

Permeability tests were conducted from April to September 2024 (170 days), with 24 columns being fed with raw leachate and another 2 with tap water (control). The temperature of leachates varied from 12.3 to 32.2 °C during the experiment. Even though such variation was significant, its effect on viscosity was considered negligible (up to ~50%) compared to the multi-order-of-magnitude reductions observed. Furthermore, all columns were exposed to the same temperature conditions, preserving the validity of relative comparisons. Upflow was adopted to guarantee the saturation and anaerobiosis within all the materials (Fig. 2). Intermittent flow was adopted to simulate real operation conditions in landfills; thus, continuous flow was adopted during about 1 h daily (simulating leachate pumping at about 0.5·m^3^/m^2^·h), and batch conditions remained during the other hours. Even though the instant flux rate was higher than the one adopted elsewhere (Rowe et al. 2002; Van Gulck and Rowe 2004), the total hydraulic and mass charge applied daily was the same (about 0.5·m^3^/m^2^·d). During continuous flow, hydraulic head and flow rate were monitored, and samples were collected for physicochemical analysis.

The parameters pH, electrical conductivity (EC), oxidoreduction potential (ORP), dissolved oxygen (DO), and temperature were monitored daily, using a multiparametric sonde HI98194. Dissolved organic carbon (DOC) (Multi N/C 2100 Analytik Jena), alkalinity (titration method), ammonium (Thermo Scientific™ Orion™ 9512 sensor), nitrate, sulfate, and chloride (Metrohm® 882 ion chromatography) were assessed bimonthly. At the end of the experiment, BART® tests were used to assess the presence of Sulfate-Reducing Bacteria (SRB), 193–223 in the influent and effluent leachates, with reactions being observed before 3 days. Such bacteria were addressed considering their potential to reduce sulfates, contributing to metals precipitation into sulfide minerals (Pérez-de-Mora et al. 2024). Finally, dissolved metals were analyzed for the initial conditions aiming to assess their leaching from the waste materials, using ICP/MS.

The results of hydraulic conductivity and physicochemical parameters for the different columns (CDW, CDW/TW, and IN CDW) were compared using analysis of variance (ANOVA) or the Kruskal–Wallis test using *PAST 5.1*.

## Results and discussion

**Fig. 3:**
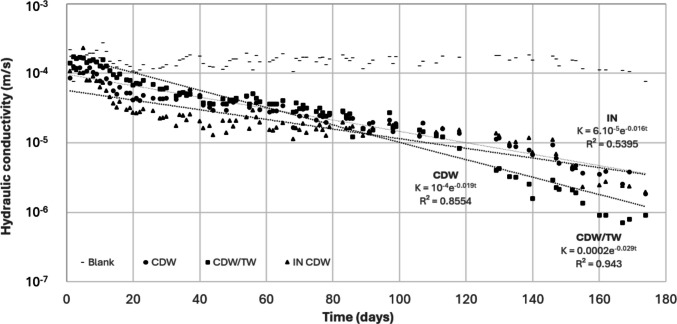
Evolution of hydraulic conductivities (average values of 8 replicates) and corresponding fitting equations for the different barrier configurations, during the experiment. The relative standard deviations for the blank, CDW, CDW/TW, and IN CDW were on average about 25, 43, 88, and 54%, respectively

**Fig. 4:**
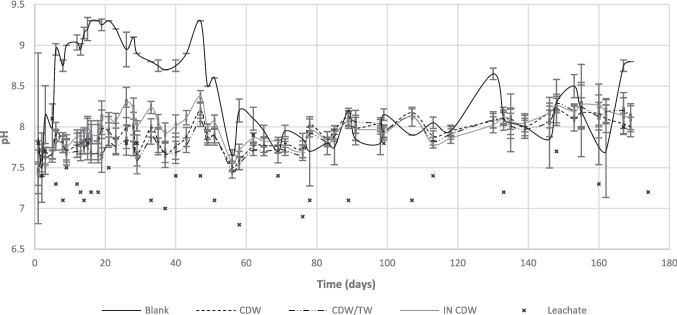
Evolution of pH values for the different barrier configurations, during the experiment

**Fig. 5:**
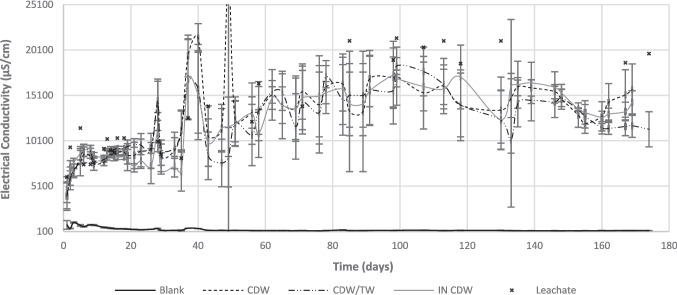
Evolution of electrical conductivities for the different barrier configurations, during the experiment

**Fig. 6:**
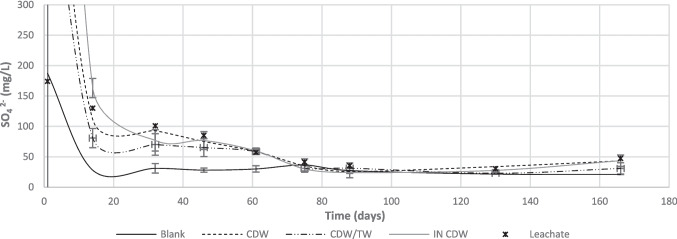
Evolution of sulfate concentrations for the different barrier configurations, during the experiment

**Fig. 7:**
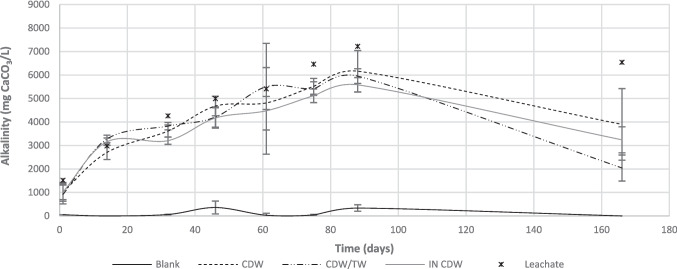
Evolution of alkalinity values for the different treatments, during the experiment

The reduction in hydraulic conductivity during the experiment is shown in Fig. 3, whereas Figs. 4, 5, 6, and 7 show the evolution of the parameters pH, EC, SO_4_^2−^, and alkalinity.

It can be observed that the starting hydraulic conductivities, as well as the one maintained for the blank columns, are approximately 10^–4^ m/s. Until approximately day 100, the lowest values were obtained for the inoculated columns (IN CDW); after that, the conductivities were similar for CDW and IN CDW columns (≈10^–6^ m/s), and the lowest values were obtained for CDW/TW columns, reaching 10^–7^ m/s in day 170 (3 orders of magnitude decrease). Such difference between barrier configurations was found to be statistically significant according to Kruskal–Wallis test (*H*^2^ = 8.304, *p* = 0.01 < 0.05).

Van Gulck and Rowe (2004), when studying columns packed with glass beads fed with synthetic leachate, obtained a 5-order of magnitude reduction after 427 days of operation at 0.54·m^3^/m^2^·day, reaching conductivities of up to 10^–6^ m/s. Clogging was attributed to a combination of biofilm growth and calcium precipitates accumulating within the pore space of the medium. Thus, it is supposed that the IN CDW columns permitted a faster development of biofilms in the beginning of the experiment, being associated with a more intense clogging. Nevertheless, after about 3 months, the bacteria naturally present in the raw leachate could colonize the surface of the particles of all barrier columns, permitting a better biofilm development and, consequently, causing a more intense reduction in hydraulic conductivity values for all studied configurations, and especially for the mixture CDW/TW.

In this regard, it is important to highlight that surface plays an important role in biofilm formation, and hydrophobic surfaces were found to be more favorable to cell immobilization and biofilm development than hydrophilic surfaces (Guo et al. 2015). As tire waste is hydrophobic (Lin et al. 2008) and cement and concrete materials, present in CDW, are hydrophilic (Liu et al. 2017), it is supposed that CDW/TW columns are more appropriate for biofilm adhesion than CDW ones. Additionally, the higher relative standard deviation observed for CDW/TW columns can be attributed to the heterogeneous and deformable nature of tire waste particles, which may promote non-uniform pore structures and localized clogging patterns.

Hydraulic conductivity values decreased more intensely after day 100 (Fig. 3). It is suggested that such period worked as an adaptation for the bacteria (lag phase), which had more intense adhesion and biofilm development afterwards. It is also important to consider the higher temperatures observed after July in Spain (after day 90), which may have contributed to a more intense microbiological development and, consequently, more clogging, for all barrier configurations.

Even having promising results, it is important to highlight that hydraulic conductivity values are still higher than the legal limit for barriers underlying inert/non-hazardous waste (10^–9^ m/s), for all studied configurations. Nevertheless, such materials could integrate a multi-barrier system with decreasing permeabilities, possibly protecting the underlying clay liners or geomembranes. Additionally, it is supposed that the materials can undergo more obstruction to reach permeabilities compatible with the legal limit. In this regard, an exponential decrease fitted well the CDW/TW data, with *R*^2^ = 0.943 for the equation *K* = 0.0002 e^−0.029t^ (Fig. 3). Considering such equation, the hydraulic conductivity of the material will reach 10^–9^ m/s after 420 days of operation, protecting the liners below the proposed barrier.

Zhang et al. (2021), when studying the effect of bio-clogging on the hydraulic conductivity of soils treated with the bacterium *Escherichia coli* and fibers, obtained reductions of several orders of magnitude, reaching values low enough for barrier applications after less than 220 h. Considering that *Escherichia coli* is naturally present in leachates, it is also considered that such bacteria—as well as other biofilm-forming species—would attach to the waste mixture, helping it achieve conductivities compatible with barrier applications.

The values of pH (Fig. 4) ranged from 7.5 to 8.5 during the greatest time of the experiment, for all barrier configurations, showing a slightly basic environment inside the columns, adequate for biofilm development (Sunil et al., 2011). The highest values were obtained for Blank samples, possibly due to the contribution of basic materials within CDW (Regadío et al. 2023). In this regard, CDW is rich in carbonates and bicarbonates, which consume protons and increase the pH values. Nevertheless, it seems that a leaching of such ions from Blank columns occurred over time, so that pH values remained moderately stable and closer to neutrality after day 60. Afterwards, all configurations presented similar results, with values close to 8.0, whereas influent leachates presented lower values during all the experiment, but also a little basic (7.0–7.5).

Finally, a tendency of pH increase seems to have occurred after day 120, to values constantly superior to 8.0, showing the occurrence of reactions that might be consuming protons from the system. One hypothesis for that is the sulfate reduction, which consumes protons, leading to an increase in environmental pH values (Tran et al. 2021). This agrees with the results of the BART® tests conducted by the end of the experiment (day 170), which showed high concentrations of SRB inside the barriers (up to 500,000 CFU/mL for CDW and IN CDW columns, and 115,000 CFU/mL for CDW/TW columns).

Additionally, it is important to mention that more heavy metals can undergo chemical precipitation at higher pH values (Pavlovic et al., 2007); thus, the contact between leachates and waste materials can be beneficial in promoting metals retention within the proposed barrier.

As for the ORP values, effluent samples showed oxic or anoxic environments (–50 to 100 mV), but not strict anaerobic ones, whereas raw leachate was always anaerobic (–250 to –50 mV). This shows that even though the system was kept closed during all the experiments, and upflow aimed to maintain saturated and anaerobic conditions within the materials, oxygenation occurred, probably in the last part of the experiments, within the sampling bottles and sampling manipulations and analyses preparation. Nevertheless, it is assumed that anaerobic conditions could still occur inside the barriers, which is confirmed by the results of the BART ® tests, in which a dense anaerobic SRB consortium was detected, but no aerobic SRB consortium was observed. In this regard, it can also be assumed that sulfate reduction can still occur within stratified environments, with associated bacteria being present in deeper zones of the biofilms (Yu and Bishop 2001).

Regarding EC (Fig. 5), the passage through the different barriers enabled a mild reduction in electrical conductivity values, of about 5000 µS/cm for all barrier configurations, showing that ions can have precipitated within the materials. By the end of the experiment, the results for CDW/TW were lower, possibly indicating that this configuration is more effective in reducing the release of contaminants to the environment. The observed differences were statistically relevant according to the Kruskal–Wallis test (*H*^2^ = 11.61, *p* = 0.003 < 0.05).

Effluent samples followed the same pattern of the influent leachate, with a mild increase over time. Such a pattern can be attributed to seasonal fluctuations in leachate quality, with an increase in temperatures and a decrease in pluviosity possibly enhancing evaporation and increasing concentrations in leachate samples. The leachate tank was refilled with newly fresh leachate every 2–3 days.

Concerning the possibility of contaminants leaching from the materials used in the barriers, the EC of Blank samples indicated that some ions seem to have been washed from the materials in the beginning of the experiment, but such process stabilized after day 40. The soluble ions washed from CDW and TW are mainly sulfate, calcium, sodium, and potassium.

Regarding SO_4_^2−^ monitoring (Fig. 6), the results showed that there was a significant release from the waste materials in the first sampling, with concentrations reaching 697 ± 365, 568 ± 254, and 1436 ± 254 mg/L for CDW, CDW/TW, and IN CDW effluents, respectively. After 15 days, however, the concentrations decreased to values < 100 mg/L, stabilizing at around 30 mg/L after day 80, for all studied configurations. The observed concentrations of effluents were mildly lower than raw leachate’s concentrations, possibly indicating sulfate consumption within the columns. Additionally, considering that blank effluents presented concentrations higher than tap water’s (< 10 mg/L), it is suggested that sulfate from the waste materials is also consumed within the columns fed with leachates. This agrees with the results of BART® tests, which showed abundance of SRB for all barrier configurations, except for the blank columns. Once again, the results for CDW/TW columns are more promising for the retention of contaminants, when compared with other configurations, not only because sulfate leaching is lower but also because this can be associated with sulfate reduction permitting metals precipitation into sulfide minerals (Pérez-de-Mora et al. 2024).

Similarly, alkalinity values of effluents from all studied configurations were lower than raw leachate’s, indicating alkalinity consumption within the barrier processes (Fig. 7). Additionally, a reduction in effluent values was observed after day 90, even leachate concentrations being kept approximately constant. This shows that alkalinity was consumed more intensely by the end of the experiment, possibly favoring metals precipitation into carbonates, bicarbonates, or hydroxides. The concentrations of effluents after passing through CDW/TW are generally lower than the other studied configurations, confirming the tendency observed for other parameters.

Regarding dissolved organic matter, DOC analysis showed that effluent concentrations were statistically equal to raw leachate ones, ranging from 150 to 450 mg/L. Thus, no depletion within the barrier was observed over time during the experiment.

With respect to metals analysis, performed only for initial conditions (days 1 and 45), blank outlet samples complied with WHO drinking standards, including Sb and Cd, which were previously considered of concern by Regadío et al. (2023). This shows that the waste materials do not seem to leach problematic contaminants to the environment. On the other hand, outlet samples from the columns fed with raw leachate also complied with the drinking standards, except for Ni and As, for which concentrations of up to 167 and 219 µg/L, respectively, were obtained, regardless of the configuration. Nevertheless, such values were below the influent leachate ones, showing a decrease in concentrations obtained within the barrier. Decreases in outlet concentrations were also observed for Cr, Co, and As, indicating their immobilization in the barrier. As previously discussed, the high pH values and the anaerobic conditions within the barrier possibly propitiated metals precipitation, as observed in predominance phase diagrams (Drever 1997).

## Conclusions

This study aimed to assess the feasibility of using construction and demolition waste and tire waste as support materials for the development of biobarriers within the landfill design. For that, PVC columns filled with the studied materials and fed with raw leachate were monitored over 170 days.

The results showed a 2–3 orders of magnitude reduction in hydraulic conductivity values after the studied period, reaching values of up to 10^–7^ m/s. The best results were obtained for the mixture of CDW and TW (50% v/v), possibly due to the hydrophobicity of tire materials permitting better adhesion of bacteria and biofilm development. An exponential equation fitted well the conductivity reduction for such a case, and values compatible with barrier applications (10^–9^ m/s) could be reached after 420 days of operation, which agrees with previous similar studies. After this period, it is assumed that the waste materials would function as clay liners, protecting them in the long term. Additionally, even in shorter periods, the obtained reduction could also help protect the liners, in a multi-barrier system composed of overlapping materials with gradual reduction of permeabilities.

The chemical monitoring showed evidence of anaerobic environments, sulfate reduction and alkalinity consumption inside the barrier, which is suggested to be beneficial to promote precipitation of metals from the raw leachates. Thus, apart from functioning as a physical barrier, the biobarriers could also work in attenuating contaminants, especially thorough biochemical precipitation and sorption processes.

The incorporation of waste materials in projected landfill liners can be beneficial to protecting the clay layers, increasing their lifetime, and potentially reducing their thickness. The proposal would contribute to reducing contamination in the medium and long term and fostering the circular economy. Given that the CDW/TW mixing ratios were not optimized in the present study, a systematic evaluation of different mixing proportions is recommended for future work.

## Data Availability

Data will be made available on request.
